# Transfemoral hepatic vein catheterization reduces procedure time in double vein embolization

**DOI:** 10.1186/s42155-024-00463-5

**Published:** 2024-05-22

**Authors:** Dominik A. Steffen, Arash Najafi, Christoph A. Binkert

**Affiliations:** Department of Radiology and Nuclear Medicine, Cantonal Hospital Winterthur, Brauerstrasse 15, Winterthur, CH-8401 Switzerland

**Keywords:** Double vein embolization, Hepatic vein embolization

## Abstract

**Background:**

Double vein embolization with simultaneous embolization of the portal and hepatic vein aims to grow the future liver remnant in preparation for major hepatectomy. Transvenous hepatic vein embolization is usually done via a transjugular access. The purpose of this study is to describe the transfemoral approach as an alternative option and to discuss potential advantages.

**Results:**

Twenty-three patients undergoing hepatic vein embolization via a transjugular (*n* = 10) or transfemoral access (*n* = 13) were evaluated retrospectively. In all cases the portal vein embolization was done first. All procedures were technically successful. There were no peri-interventional complications. Only two patients were not able to proceed to surgery. Standardized future liver remnant hypertrophy was non-inferior with the transfemoral approach compared to the transjugular route. Procedure time was significantly shorter in the transfemoral access group (40 ± 13 min) compared to the transjugular group (67 ± 13 min, *p* < 0.001).

**Conclusion:**

Transfemoral hepatic vein embolization is feasible, safe, and faster due to easier catheterization, improved stability, and simpler patient preparation. These findings will need to be validated in larger studies.

## Background

Combined portal and hepatic vein embolization improves the resectability of primary hepatic malignancies and liver metastases by inducing hypertrophy in the future liver remnant (FLR) and thus reducing the risk of post-hepatectomy liver failure [1–4]. While the portal vein is generally embolized with liquid embolic agents via a percutaneous transhepatic approach, two different techniques have emerged for hepatic vein embolization. During double vein embolization (DVE), the hepatic veins are embolized with plugs from a transjugular approach, whereas in liver venous deprivation the hepatic veins are embolized with plugs and glue via a transhepatic puncture [5–7]. The two methods have recently been shown to be comparable regarding safety and effectiveness [8]. In this study, we describe a technical variation of DVE using a transfemoral venous approach.

## Materials and methods

Patients undergoing double vein embolization at a single tertiary center between 2017 and 2023 were retrospectively reviewed and grouped by access route used for hepatic vein embolization. Indication for DVE was based on preoperative FLR volume, presence of liver disease, history of chemotherapy, and extent of planned liver surgery. The treatment strategy was determined by consensus in a multidisciplinary tumor board. Written informed consent was obtained from all patients and the study was approved by the local ethics committee. The embolization procedures were conducted by four interventionalists with 2–16 years of experience in endovascular procedures.

### Embolization technique

Portal vein embolization was performed first via an ipsilateral approach using a mixture of N-butyl cyanoacrylate (Glubran II, GEM, Viareggio, Italy) and iodized oil (Lipiodol, Guerbet, Aulnay-sous-Bois, France). The dilution varied between 1:1 to 1:5 depending on operator preference.

For transvenous hepatic vein embolization, ultrasound-guided access to either the right internal jugular or the right common femoral vein was used. A 6.7 F multipurpose angled tip sheath (Vista Brite Tip, Cordis, Miami Lakes, FL) was used for the transjugular approach and a 6.7 F renal double curve (RDC) guiding sheath (Vista Brite Tip, Cordis, Miami Lakes, FL) for the transfemoral approach, respectively. When necessary, a coaxial catheter was used to achieve a peripheral catheterization. Both central and peripheral branches of the right hepatic vein were occluded using Amplatzer type II and IV plugs (Abbott Laboratories, North Chicago IL). In select cases, the middle hepatic vein and accessory veins of liver segment VI were also embolized in the same manner. Indication for accessory vein embolization was based on vessel size and drained liver volume as assessed on pre-interventional CT.

### Parameters and statistical analysis

The procedure time was measured from imaging documentation, i.e. the interval between the first and the last image of the procedure. The portal vein embolization part of DVE was subtracted from this measurement because the present study focused on the hepatic vein embolization part of DVE and portal vein embolization duration can vary greatly depending on technique and ease of access. Accessory liver veins were identified on preprocedural CT. The embolization rate of accessory liver veins and the number of plugs were recorded from intraprocedural documentation. CT liver volumetry was performed on all patients before and within 30 days of DVE. Volumetric parameters were calculated as described in literature [2, 7–10]. 

All statistical analyses were performed using R 4.3.2 (R Foundation for Statistical Computing, Vienna, Austria). Values are expressed as mean ± standard deviation. The Mann-Whitney U test, Fisher’s exact test and the chi-square test were used for comparative statistics. A *p*-value < 0.05 was considered statistically significant.

## Results

**Fig. 1:**
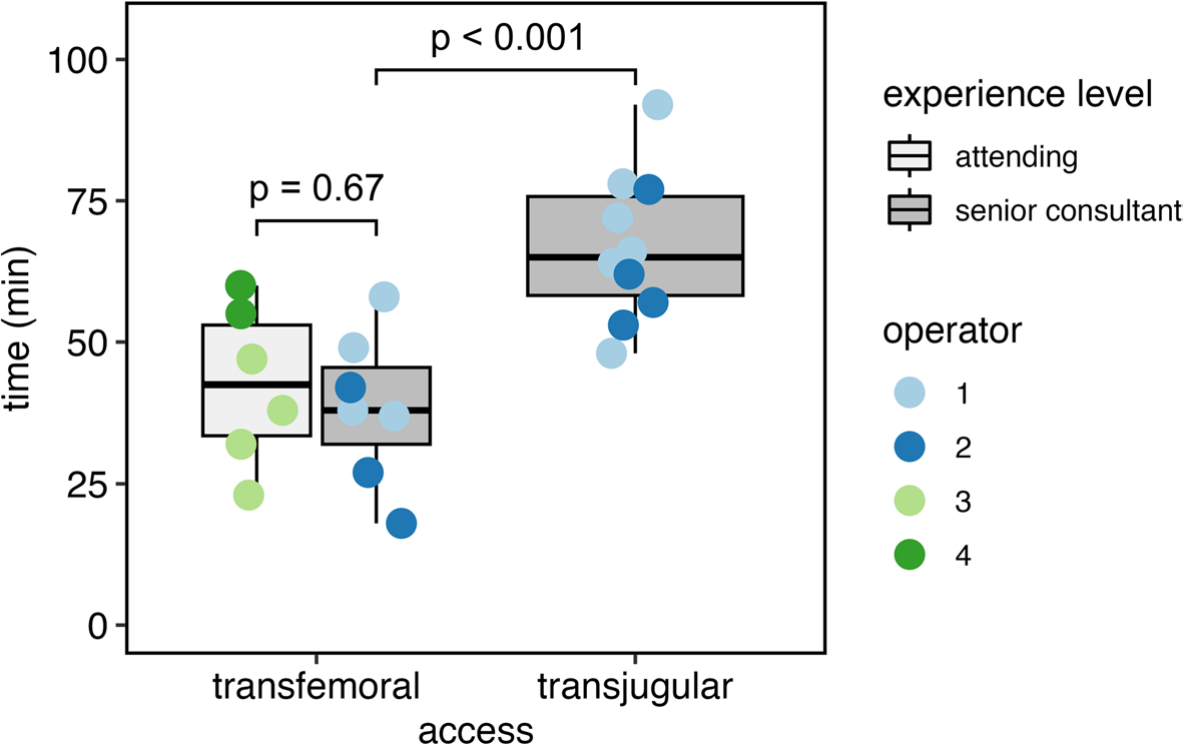
Procedure times grouped by access route and operator experience. Procedure length was significantly shorter (*p* < 0.001) when using the transfemoral approach, independent of operator experience (*p* = 0.67)

Twenty-three patients underwent DVE through either a transjugular (*n* = 10) or transfemoral (*n* = 13) venous approach for hepatic embolization. Patient characteristics are given in Table 1. All procedures were technically successful. There were no peri-interventional complications. One patient in the transjugular group suffering from cholangiocarcinoma died 40 days after the intervention due to infected tumor necrosis and sepsis. One patient in the transfemoral group did not proceed to surgery due to extrahepatic tumor progression. All other patients exhibited sufficient liver hypertrophy and underwent surgery. Patients’ baseline and follow up volumetric data are given in Table 2. A higher degree of hypertrophy was observed in the transfemoral group (16.9% ± 6.8 vs. 10.7% ± 5.3, *p* < 0.01). Mean procedure time was 40 ± 13 min for the transfemoral approach and 67 ± 13 min for the transjugular approach, respectively (*p* < 0.001, Fig. 1). For the transfemoral approach, procedure time was independent of operator experience (*p* = 0.67). Four out of seven accessory veins were embolized in the transfemoral group and 3/7 in the transjugular group (*p* = 1). The middle hepatic vein was embolized in one patient in the transfemoral group and in three patients in the transjugular group (*p* = 0.28). The number of plugs used did not differ between the groups (5 ± 1.78 for transfemoral and 5.5 ± 1.58 for transjugular, *p* = 0.48).

**Table 1:** Patient characteristics

	**Transjugular approach (** ***n*** **=10)**	**Transfemoral approach (** ***n*** **=13)**	***p*** **-value**
Age (y)	63.1 ± 14.2	59.6 ± 9.3	0.3
Male gender (n)	7 (70%)	9 (69%)	1
Tumor type (n)			0.65
Metastases	7 (70%)	9 (69%)	
Cholangiocarcinoma	3 (30%)	3 (23%)	
Hepatocellular carcinoma	0 (0%)	1 (8%)	
Cirrhosis (n)	0 (0%)	1 (8%)	1
Neoadjuvant chemotherapy (n)	6 (60%)	10 (77%)	0.65
Proposed type of surgery (n)			0.17
Hemihepatectomy	3 (30%)	5 (38%)	
Hemihepatectomy + wedge resection	1 (10%)	5 (38%)	
Extended hemihepatectomy	4 (40%)	3 (23%)	
Extended hemihepatectomy + wedge resection	2 (20%)	0 (0%)	

**Table 2:** Liver volumetry

	**Transjugular approach (** ***n*** **=10)**	**Transfemoral approach (** ***n*** **=13)**	***p*** **-value**
FLR pre-interventional [ml]	632 ± 214	545 ± 216	0.26
FLR post-interventional [ml]	797 ± 193	821 ± 243	0.97
FLR increase (%)	30.2 ± 19.5	56.6 ± 24.1	0.02
sFLR pre-interventional (%)	41.7 ± 17.4	32.8 ± 12.2	0.26
sFLR post-interventional (%)	52.4 ± 17.1	49.7 ± 14.9	0.79
Degree of hypertrophy (%)	10.7 ± 5.3	16.9 ± 6.8	<0.01
Follow up scan delay (d)	14.7 ± 8.3	14 ± 0.4	1

$$FLR\;increase=\frac1n{\textstyle\sum_i^n}\frac{FLR_{post,i}-FLR_{pre,i}}{FLR_{pre,i}}\times100\%$$ [8]

$$sFLR=\frac{FLR}{-794+1267\times BSA}$$ [9, 10]

$$degree\;of\;hypertrophy\;(\%)=sFLR_{post}\;(\%)-sFLR_{pre}\;(\%)$$ [2, 7]

## Discussion

**Fig. 2:**
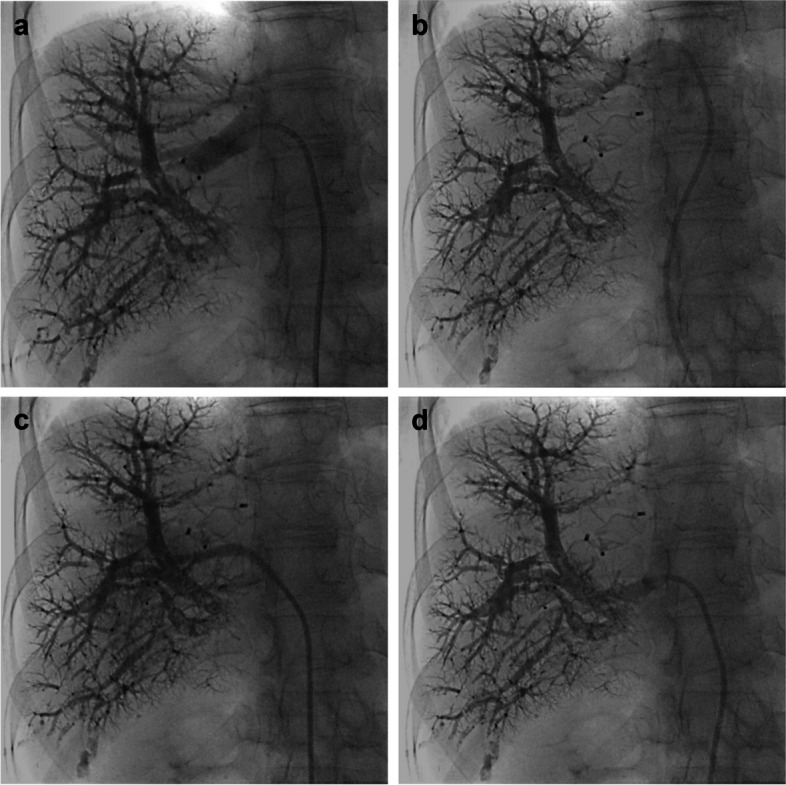
RDC guiding sheath positioned in the main right hepatic vein (**a**), a superior branch (**b**) and two separate accessory inferior veins (**c **and** d**)

We first used the transfemoral access in a patient with a horizontal segment VI vein which was deemed inaccessible from a jugular access. Using the femoral approach, catheterization was easy and the sheath was stable during plug insertion. Due to the experience of this initial and subsequent cases, we transitioned to a femoral first approach for DVE. Using this method, the two less experienced operators had near-identical procedure times as their senior colleagues despite having no prior training in transjugular DVE (Fig. 1). In our experience, transfemoral access offers several advantages in terms of usability. Apart from the facilitated catheterization of acutely angled accessory hepatic veins, respiratory motion tends to be less of a problem as the double curve configuration of the RDC sheath stabilizes it against the vessel wall (Fig. 2). Notably, femoral access also obviates the need to reposition the patient’s arm after successful transhepatic portal vein embolization to allow access to the neck. Sequential preparation of a second sterile field is not required either as the right groin and the right hemiabdomen can be disinfected in one contiguous field. One potential drawback of the femoral puncture site is the need for a pressure dressing and possibly increased bleeding rates, though we did not experience any such events in our cohort.

These benefits are reflected in reduced procedure time (40 ± 13 min vs. 68 ± 13 min). Further, significantly higher FLR growth was observed in patients with transfemoral hepatic embolization. Since the parameters analyzed in our cohort which focused on the hepatic vein embolization part of DVE do not provide an explanation for this, the observed difference in FLR growth may be due to differences in the portal vein embolization technique or patient-related factors other than those evaluated in our study. Therefore, no conclusions of superiority should be drawn from this observation. It seems safe to assume that transfemoral embolization is non-inferior to transjugular hepatic vein embolization in terms of liver hypertrophy, but this requires validation in larger studies.

## Conclusion

Transfemoral approach for hepatic vein embolization in DVE is feasible and equally safe and effective as the transjugular approach. In our experience, easier catheterization, improved stability, and simplified patient preparation reduce the duration of the procedure. We have therefore changed our practice to a femoral first approach for DVE.

## Data Availability

The datasets analyzed during the current study are available from the corresponding author on reasonable request.

## Abbreviations

DVE: Double vein embolization
FLR: Future liver remnant
RDC: Renal double curve
sFLR: standardized future liver remnant
